# The impact of dental treatment and age on salivary cortisol and alpha-amylase levels of patients with varying degrees of dental anxiety

**DOI:** 10.1186/s12903-019-0901-7

**Published:** 2019-09-06

**Authors:** Majed AlMaummar, Huda Othman AlThabit, Sharat Pani

**Affiliations:** 1 National Guard Health Affairs, Riyadh, Saudi Arabia; 2 Resident in Pediatric Dentistry, Saudi Board in Pediatric Dentistry, Riyadh, Saudi Arabia; 3 Room No 1012, Schulich School of Medicine and Dentistry, Western University, London, ON Canada

**Keywords:** Alpha-amylase, Anxiety, Cortisol, Dental fear, Salivary biomarkers

## Abstract

**Background:**

The purpose of this study was to assess the salivary cortisol and salivary alpha-amylase levels in children aged between 6 and 9 years, 3 months and 1 year after the successful completion of dental treatment through either pharmacological or non-pharmacological behavior management techniques.

**Methods:**

A total of 1567 patients aged between 6 and 9 years who had completed dental treatment were screened. A total of 703 patients who were caries free at the end of 3 months were classified based on Frankl behavior score and administered the Arabic version of the Children’s Fear Survey Schedule- Dental Subscale (CFSS-DS) and accordingly allocated to one of three groups; (Phobic Patients, Anxious Patients, Control Group). A total of 183 patients met the inclusion criteria and were followed up for 1 year. A total of 151 patients completed the study. Patients’ heart rate on recall, salivary cortisol and salivary amylase were compared between the groups.

**Results:**

The results of the study showed that amylase and cortisol levels had a significant association with the level of dental fear. The phobic patients had the highest levels of salivary amylase and salivary cortisol levels with no significant associations observed with either heart rate or extent of dental treatment. Control and anxious patients had significantly lower amylase levels when compared to phobic patients. There was no significant difference between the salivary cortisol levels of anxious and phobic patients. These findings were replicated on 1-year recall.

**Conclusions:**

Within the limitations of this study we can conclude that salivary amylase is an indicator of of acute stress that can differentiate between anxiety and dental fear; while salivary cortisol appears to be a marker of long-term stress that lacks the sensitivity to differentiate between the two.

## Background

Dental fear remains a significant challenge to obtaining good oral healthcare with multiple studies showing association between dental fear and reduced dental visits [1–3]. However, the study of fear remains, to a large extent, based on subjective analyses and questionnaires.

Specific phobia is defined by the Diagnostic and Statistical Manual for mental disorders (DSM- V) as being fearful or anxious about, or avoidant of, circumscribed objects or situations [4] . The DSM- V classifies dental phobias as a subtype of a specific phobia termed as blood-injection-injury (BII) [4]. Despite being regularly reported in literature there remains difficulty in accurately diagnosing dental phobia and differentiating it from anxiety and fear [5].

There have been attempts made to study the development of dental phobia and the change in phobic behavior according to the age of the child [6–8]. Researchers agree that there is a strong relationship between dental fear and the normal cognitive and psychological development of individuals [9, 10]. Children aged between 6 and 9 years have begun the process of cognitive development and exhibit both anxiety and phobia. Behavioral scientists have therefore used this age group previously for the analysis of phobias and behavioral problems [11, 12].

Salivary cortisol levels provide an accurate, reliable and non-invasive measure of stress in both adults and children [13]. Cortisol is a hormone secreted by the hypothalamus pituitary adrenal axis (HPAA) and has been used an accurate biomarker in stress research for over half a century [14]. In dentistry salivary cortisol has been used to measure the role of stress in the anxiety of dental treatment [15, 16]. Research suggests that salivary alpha-amylase may serve as a complementary diagnostic tool to salivary cortisol. While cortisol is seen as a predictor of long term stress, research suggests that salivary alpha amylase may offer more sensitive readings of short term stress [17–19].

The use of salivary hormones raises certain temporal and situational issues and diurnal variations in the levels of hormones, especially cortisol are well documented [15, 16, 19]. To overcome these the use of midmorning saliva and protocols for the collection and storage of saliva have been developed [14, 20].

While biomarkers are a useful indicator of long term stress, heart rate is a far more useful indicator of immediate fear, anxiety and stress [21]. While there have been studies that use salivary markers as biomarkers for dental fear, phobia as a specific condition has received less attention. Only few studies have actually attempted to differentiate between phobic patients and those who are anxious and have been successfully managed through behavior modification [22]. The aim of this study was to evaluate salivary cortisol and alpha amylase levels in patients who are not afraid of the dentist, patients who have overcome their fear of the dentist through behavior management and patients who cannot be managed by non-pharmacological behavior management. The study also aimed to evaluate the salivary cortisol and alpha amylase levels in patients before and after behavior management and to correlate these readings to the outcome of the behavior management.

## Methods

### Ethical approval

The study was registered with the research centers of the King Abdullah International Medical Research Center (KIAMRC), Riyadh, Saudi Arabia and the Institutional Review Board of the Riyadh Elm University, Riyadh, Saudi Arabia. Ethical approval was obtained from the Institutional Review Board of the KIAMRC (RC15–007). Written informed consent was obtained from all parents/guardians of the children and verbal assent was obtained from the children prior to examining the child and/or collecting salivary samples.

### Source of patients

The treatment records of patients treated at the dental clinics of King Abdulaziz Medical City, and the College of Dentistry, Riyadh Elm University, Riyadh, Saudi Arabia were screened to locate patients who had successfully completed dental treatment. A total of 1567 records of children aged between 6 and 9 years were screened for the behavior as recorded in the chart. Behavior screening was done using the Frankl behavior rating scale where by patients were rated on a four point scale; definitely negative (−-), negative (−), positive (+) and definitely positive (++).Patients were allocated to one of three groups;

Control Group (Group A) comprised patients who had been given a Frankl score of positive (+) or definitely positive (++) on the first dental visit and had completed their dental treatment after successful behavior management which included a recommended protocol of tell show do, followed by administration of the dental treatment. These patients were recalled 3 months after the completion of treatment and administered an Arabic version of the CFSS-DS. Patients who scored lower than 20 on the recall visit were assigned to this group.

Anxious Patients (Group B): comprised patients who had been given a Frankl score of negative (−) or definitely negative (−-) on the first dental visit but had completed their dental treatment after successful behavior management, which included the combined use of tell show do, modelling and/or voice control. These patients were recalled 3 months after the completion of treatment and administered an Arabic version of the CFSS-DS. Patients who scored higher than 32 on the recall visit were assigned to this group.

Phobic Patients (Group C): comprised patients who had been given a Frankl score of definitely negative (−-) on two separate occasions and had completed their dental treatment under general anesthesia (GA). These patients were recalled 3 months after the completion of treatment and administered an Arabic version of the CFSS-DS. Children who scored above 32 on the CFSS-DS in the recall visit were classified as phobic patients using previously proposed criteria.

**Fig. 1:**
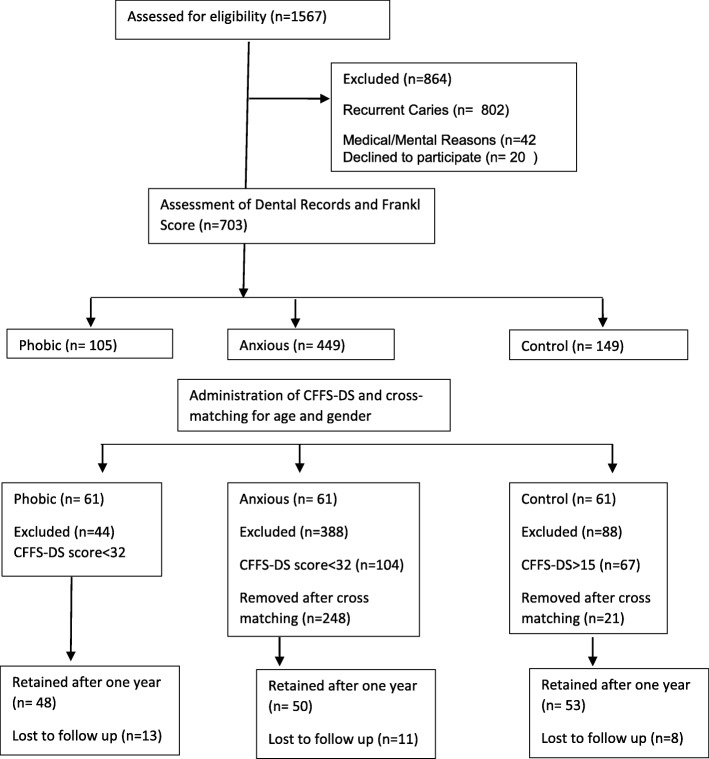
Selection and Distribution of the study population

Patients with recurrent caries on the recall visit, patients with history of chronic illness or mental illness were excluded from the study. Out of the 1567 records screened a total of 183 patients met the initial inclusion and cross matching criteria. After 1 year a total of 32 patients were lost to follow up (Fig. 1).

### Sample size and power

The sample power was calculated using the G power Sample size calculator (Universtat Kiel, Keil Germany). Multiple sample power protocols were calculated for the one-way ANOVA and the Kruskull Wallis test. The protocol showed that the minimum required subjects for an alpha of 0.05 with (1-β) of 0.95 and effect size of 0.5 (large effect size) was 66. The final sample achieved (*n* = 153) gave an actual power of 0.987.

### Data collection

#### Collection and analysis of saliva

Stimulated saliva was collected using the passive drool method [23] into sterile collection tubes (Greiner Bio-One GmbH, Austria). These tubes were stored in a vaccine box (Apex International, Uttar Pradesh, India) at ≈5 °C for half an hour before being transferred to a deep freezer at -80 °C where they were stored until analysis. Early morning saliva was collected 3 hours after the patient wakes up, noon saliva was collected at 12 pm and evening saliva was collected at 8 pm. The saliva cortisol and amylase levels were then normalized for diurnal variation using standard protocols [13, 14].

The salivary cortisol was measured using commercially available chemoimmunoluminiscence assay kits for salivary amylase and salivary cortisol (Cobas integra400 plus, Roche Diagnostics, Risch-Rotkreuz, Switzerland) and analyzer (Cobas e411, Roche Diagnostics, Risch-Rotkreuz, Switzerland).

#### Monitoring of the heart rate

Heart rate was measured using a pulse oximeter (Beurer, PO30, Beurer GmbH, Uttenweiler, Germany) and recorded in beats per minute (bpm). Heart rate was recorded in the waiting room prior to the commencement of treatment. Heart rate was recorded over a 5-min period at 30 s intervals and the mean heart rate was used for the analyses.

#### Administration of Questionnaire and Data Collection

The demographic data of the child was collected by administering a questionnaire to the father and the mother. The questionnaire contained questions regarding the age, sex, taking other medication, The next part of a questionnaire filled by the dentist during the clinical examination contained Behavior management, Pulse rate, Number of carious teeth, Number of recurrent carious teeth, Plaque accumulation, Number of SSC,Recall visit by month. The last part consisted of the CFFS-DS questionnaire that was administered to the child. The Children’s Fear Survey Schedule- children’s subscale (CFSS-DS) is a subjective measure of dental fear that has been validated in Arabic and has been found to be useful in the measurement of fear in children [24]. The tool has been previously used to differentiate between fearful and phobic dental patients. The questionnaire also served as a means of obtaining informed consent.

### Statistical analyses

Given the predisposition of salivary biomarkers to exhibit skew, an analysis of skew was done on the cortisol and amylase values of the population. No significant skew was found in the distribution of either cortisol (skew = 0.12) or amylase (skew = 0.09), thus allowing for the use of parametric statistics. The values of salivary cortisol and amylase were compared between the groups using a one-way ANOVA and Tukey’s post hoc test. Gender differences were analyzed using the t test. All statistical analyses were carried out using the SPSS ver.21 data processing software (IBM Corp, Armonk, NY).

## Results

Of the 1567 records screened at the dental clinics of King Abdulaziz Medical City, and the Riyadh Colleges of Dentistry and Pharmacy in Riyadh a total of 151 patients completed the recall evaluation. There were 77 girls and 74 boys with a mean age of 7.43 (SD = 1.2). The patients were aged between 6 years and 9 years and though the males were slightly older than the females, there was no significant difference in age between the genders (*p* = 0.089). Although, there were more females (*n* = 77) than males (*n* = 74) the chi square test showed these differences to be statistically insignificant.

The average heart between different groups was 97.3 (SD = 13.77) with the highest heart rates observed with anxious patients. The one-way ANOVA showed these differences to be significant at both the 3 month and the 1-year findings (Table 1). The Tukey’s post hoc test showed that significant differences existed between the control and the anxious patients (*p* < 0.05). Interestingly no significant differences existed between the control and the anxious patients at 3 months (*p* = 0.115) or at the 1 year follow up visit (*p* = 0.130. Similarly, no statistically significant difference between the anxious and the phobic patients was observed at 3 months (*p* = 0.404) or at 1 year follow up (*p* = 0.089).

**Table 1:** Mean heart rate (bpm) across different groups at 3 month recall and 1 year recall

Measurement time	Group	N	Mean	Std. Deviation	F	Sig*
3 months	Control^a^	61	91.53	11.14	5.547	0.005**
	Anxious^ab^	61	102.40	16.55		
	Phobic^b^	61	98.15	11.03		
1 Year	Control^a^	52	92.35	12.22	4.382	0.008**
	Anxious^ab^	50	106.11	14.53		
	Phobic^b^	48	99.12	13.21		

*Calculated using the one-way ANOVA

**Differences significant at *p* < 0.05

^a,b^ Groups with different superscripts show significant difference at *p* < 0.05 when compared using the Tukey’s post-hoc test

When the salivary amylase levels between the among different groups was tabulated at 3 months it was observed that the phobic patients had the highest levels of salivary amylase, followed by the anxious patients with the control group having the lowest levels of salivary amylase. The one-way ANOVA found that these differences were statistically significant (*p* = 0.029). The findings were replicated at the 1 year follow up visit, and although the amylase levels of the anxious patients showed some reduction, the overall difference among groups remained statistically significant (Table 2). When the differences between groups was compared using the Tukey’s post hoc test it was observed that phobic patients had significantly higher amylase levels when compared to control and anxious patients at both the 3 month and 1 year recall periods(*p* < 0.05). No significant differences were observed between the anxious patients and the control patients at both 3 month (*p* = 0.597) or 1 year (*p* = 0.492) recall measurement.

**Table 2:** Mean salivary amylase (pcg/l) levels across different groups at 3 month recall and 1 year recall

Measurement time	Group	N	Mean	Std. Deviation	F	Sig*
3 months	Control^a^	61	36,381.83	2832.02	3.693	0.029**
	Anxious^ab^	61	48,540.68	4943.94		
	Phobic^b^	61	69,986.58	2302.06		
1 Year	Control^a^	52	36,212.35	1992.72	4.082	0.007**
	Anxious^a^	50	47,206.11	2014.83		
	Phobic^b^	48	71,949.42	1813.21		

*Significance calculated using one way ANOVA

** Differences significant at *p* < 0.05

^a,b^ Groups with different superscripts show significant difference at p < 0.05 when compared using the Tukey’s post-hoc test

The salivary cortisol levels varied significantly across groups. The control group had the lowest levels of salivary cortisol while the patients classified as phobic had the highest levels of salivary cortisol (Table 3). The one-way ANOVA showed that there was a significant difference among the groups at both 3 months and 1 year post treatment (Table 3). The Tukey’s post hoc demonstrated that the cortisol levels of the control group were significantly lower than those of the anxious and phobic patients (p < 0.05). There was no significant difference between the salivary cortisol levels of anxious and phobic patients at either the 3 month follow up (*p* = 0.212) or at the 1 year follow up (*p* = 0.126).

**Table 3:** Mean salivary cortisol levels (pcg/l) across different groups at 3 month recall and 1 year recall

Measurement time	Group	N	Mean	Std. Deviation	F	Sig*
3 months	Control^a^	61	5.0434	3.053	3.693	0.000**
	Anxious^b^	61	9.8059	7.063		
	Phobic^b^	61	12.6056	4.813		
1 Year	Control^a^	52	5.6434	1.753	4.082	0.000**
	Anxious^b^	50	10.9059	6.883		
	Phobic^b^	48	11.6056	2.713		

*Significance calculated using one way ANOVA

** Differences significant at *p* < 0.05

^a,b^ Groups with different superscripts show significant difference at p < 0.05 when compared using the Tukey’s post-hoc test

## Discussion

Child dental fear is one of the major problems that dentists face in practice and has been linked to poor dental health [1, 6, 7]. Previous studies have shown that dental fear and the behavioral problems it causes are linked to the age of the child [25–27]. It is therefore reasonable that any study that aims to study dental fear must focus on a fixed age group. The age group 6–9 years has been used by several previous authors and has been proposed as an age where cognitive development begins to manifest itself [28].

Demographically this study found more females than males in the anxious group, a finding that is supported by previous studies [29, 30]. This is however contrary to other studies done on school children which have reported no difference in anxiety between girls and boys [25–27]. This study found no significant difference in age across which could be attributed to the strong age matching that was done during the stage of cross matching. The fact that we found no age difference in fear among the different groups of this study seems to validate our rationale of choosing the age of 6-9 years as a homogenous study group.

The rationale for choosing heart rate as an indicator was based on previous studies which have demonstrated its usefulness in measuring the degree of stress and anxiety in patients undergoing dental treatment [31–33] . Our findings of significant differences in measurements of heart rate between the control and the anxious patients, in the waiting area prior to dental treatment, seem to confirm that patients who are anxious face increases in heart rate even when not in the dental chair. Interestingly the patients classified as phobic using the CFSS-DS did not have a significantly higher heart rate than the control group. These findings need to be interpreted with caution, as the current study only observed the dentist’s perception of the child’s behavior and not the actual subjective feelings of the child. Furthermore the current study measured heart rate in the waiting area, unlike the study by Wannemueller et al. which looked at the heart rate in response to a specific stimulus [22].

Our rationale in choosing children 3 months after dental treatment was to abolish any possible confounding factor of dental problems. Furlan et al., showed that salivary cortisol and alpha-amylase levels (salivary biomarkers) and heart rate in children undergoing a minor dental procedure (dental prophylaxis) were not influenced by treatment [31]. The same study also showed that heart rate was the only significant predictor of acute dental stress [31].

Salivary biomarkers have been proposed as a valuable tool for evaluating anxiety-producing events, such as dental treatment, in children [34]. Our decision to use salivary alpha amylase (sAA) as a biomarker in this study was based on previous research which has shown that stress causes a significant increase in sAA levels [18, 35].

Previous studies demonstrated a positive correlation between stress, anxiety, and salivary cortisol levels [36, 37]. We found that while the salivary cortisol levels seemed to increase with the fear score there was no significant difference between patients classified as anxious and those classified as phobic. This seems to suggest that while amylase is an indicator of fear cortisol may be an indicator of anxiety. However, attempts to correlate cortisol levels to chronic anxiety have given variable results. One of the limitations of using salivary cortisol as a biomarker is the large number of confounding factors. Furthermore, existing literature as often failed to discern between acute fear and chronic anxiety.

Benjamins et al. showed that salivary free cortisol concentrations were significantly elevated if the patients manifested anxiety according to the scores on the Dental Anxiety Scale (DAS) [38]. More recent work in adults however has found no significant correlation between the DAS score and salivary cortisol levels [39]. One of the limitations of the current study is that unlike the previously mentioned work the final behavior of the child was measured using the Frankl scale, an objective measurement that doesn’t make allowance for the subjective fear experience by the child.

Our results demonstrated that the cortisol levels of the control group were significantly lower than those of the anxious and phobic patient. However, we found no significant difference between the salivary cortisol levels of anxious and phobic patients. This further validates the theory that expression of fear is a result of the activation of the sympathetic response [22].

In using the CFFS-DS as tool for measuring the extent of fear and phobia we were building on the work of El-Housseiny et al. [24] and TenBerge et al. [26]. The salivary biomarker findings of this study indicate that there is some merit to classifying patients as anxious or phobic based on their fear score. The amylase and cortisol levels seen in our patients seem to demonstrate that not only is dental phobia distinct from dental fear and/or anxiety, but it also manifested differently in terms of biomarkers secreted. The key necessity of any successful biomarker is stability. The one-year findings of our study showed that both salivary cortisol and salivary amylase; though different in what they measured, were able to provide a stable reading that was not significantly altered with time.

The current study was limited by a lack of standardization of behavior management techniques and initial behavior rating. Furthermore, the use of the Frankl rating score as an initial screening tool instead of a more specific scale was based on the need to access the records of the treating clinicians. In order to obtain the sample desired, we had to forgo calibration and/or standardization of the behavior diagnosis and management technique used. However, in order to overcome this the screening of dental anxiety was done by a single calibrated examiner (HA) and in a setting removed from the dental clinic.

An alternate method to improve calibration has been the pooling of data into fearful and non-fearful groups, thus avoiding the challenges of defining the difference between phobic and anxious patient (19,31,33). However, the current study aimed to establish that patients who score differently on anxiety scales exhibit distinct profiles of salivary cortisol and salivary amylase. The findings of this study would need to be expanded upon by future researchers to further explore the link between the two.

Despite these limitations, the results of the study provide clinical implications for researchers who seek to apply behavior modification techniques on children with dental phobia and dental anxiety. The potential of salivary cortisol and salivary amylase as biomarkers for stress research in controlled exposure studies could be the focus of further studies.

## Conclusion

Within the limitations of the study we can conclude that there is a definite relationship between dental fear and assays ofsalivary cortisol and salivary amylase in children aged between 6 years and 9 years. In this age group salivary cortisol seems to serve as an assay of dental fear but cannot differentiate between patients who are anxious and those who are phobic. Salivary amylase has poor correlation with anxiety scores but appears to be effective at detecting dental phobia in children aged between 6 years and 9 years.

## Data Availability

Data and additional material will be made available on request from the authors.

## Abbreviations

BII: Blood Injection Injury
bpm: Beats per Minute
CFSS-DS: Children’s Fear Survey Schedule- Dental Subscale
DAS: Dental Anxiety Scale
DSM- V: Diagnostic and Statistical Manual for mental disorders
GA: General Anesthesia
HPAA: Hypothalamus Pituitary Adrenal Axis
sAA: Salivary Alpha Amylase
